# Sclerotinia minor Endornavirus 1, a Novel Pathogenicity Debilitation-Associated Mycovirus with a Wide Spectrum of Horizontal Transmissibility

**DOI:** 10.3390/v10110589

**Published:** 2018-10-27

**Authors:** Dan Yang, Mingde Wu, Jing Zhang, Weidong Chen, Guoqing Li, Long Yang

**Affiliations:** 1 The State Key Laboratory of Agricultural Microbiology and Hubei Key Laboratory of Plant Pathology, Huazhong Agricultural University, Wuhan 430070, China; danyang@mail.hzau.edu.cn (D.Y.); mingde@mail.hzau.edu.cn (M.W.); zhangjing1007@mail.hzau.edu.cn (J.Z.); guoqingli@mail.hzau.edu.cn (G.L.); 2 U.S. Department of Agriculture, Agricultural Research Service, Washington State University, Pullman, WA 99164, USA; w-chen@wsu.edu

**Keywords:** Sclerotinia minor, endornavirus, hypovirulence, transmissibility, biological control

## Abstract

Sclerotinia minor is a phytopathogenic fungus causing sclerotinia blight on many economically important crops. Here, we have characterized the biological and molecular properties of a novel endornavirus, Sclerotinia minor endornavirus 1 (SmEV1), isolated from the hypovirulent strain LC22 of *S. minor*. The genome of SmEV1 is 12,626 bp long with a single, large open reading frame (ORF), coding for a putative protein of 4020 amino acids. The putative protein contains cysteine-rich region (CRR), viral methyltransferase (MTR), putative DEXDc, viral helicase (Hel), and RNA-dependent RNA polymerase (RdRp) domains. The putative protein and the conserved domains are phylogenetically related to endornaviruses. SmEV1 does not contain a site-specific nick characteristic of most previously described endornaviruses. Hypovirulence and associated traits of strain LC22 and SmEV1 were readily cotransmitted horizontally via hyphal contact to isolates of different vegetative compatibility groups of *S. minor*. Additionally, SmEV1 in strain LC22 was found capable of being transmitted vertically through sclerotia. Furthermore, mycelium fragments of hypovirulent strain LC22 have a protective activity against attack by *S. minor*. Taken together, we concluded that SmEV1 is a novel hypovirulence-associated mycovirus with a wide spectrum of transmissibility, and has potential for biological control (virocontrol) of diseases caused by *S. minor*.

## 1. Introduction

Mycoviruses or fungal viruses are viruses that can infect fungi and Oomycetes, and can replicate in these organisms [1]. They exist in all major taxonomic groups of fungi and Oomycetes [2]. As intracellular and molecular parasites, mycoviruses completely depend on their hosts for replication and transmission. Therefore, the mycoviruses have evolved various subtle and well-regulated relationships with their hosts. Previous studies showed that most mycoviruses have little or no detectable effects on the morphology and/or physiology of their hosts, and this kind of infection is usually called cryptic (or latent) infection [1]. However, a few mycoviruses do have evident effects on the morphology and/or physiology of their hosts. The beneficial effects on the hosts include increase of virulence (hypervirulence) in some plant pathogenic fungi or oomycetes [3,4], and improvement of competitive ability of some yeasts by producing killer proteins [5].

The detrimental effects on their hosts include virus diseases on cultivated mushrooms [6] and reduction of virulence (hypovirulence). So far, mycovirus conferring (or associated with) hypovirulence has been reported in many plant pathogenic fungi, including *Botrytis* spp., *Cryphonectria* (=*Endothia*) *parasitica*, *Fusarium graminearum*, *Ophiostoma novo-ulmi*, *Rhizoctonia solani*, *Rosellinia necatrix*, and *Sclerotinia* spp. [7]. Some mycoviruses can be potentially exploited for controlling plant fungal diseases. In this aspect, a well-known example is the use of Cryphonectria hypovirus 1 (CHV1) to control chestnut blight caused by *C. parasitica* [8,9]. Recently, Yu and colleagues [10] reported that Sclerotinia sclerotiorum hypovirulence-associated DNA virus 1 (SsHADV-1) in strain DT-8 of *S. sclerotiorum* can effectively suppress Sclerotinia stem rot of oilseed rape (*Brassica napus*) caused by virulent strains of *S. sclerotiorum* when the hyphal fragments of DT-8 or the virus particles of SsHADV-1 were applied on the plants. These successful examples have inspired many other researchers to search for mycoviruses with a promising biocontrol potential.

Previous studies showed that efficient transmission of the hypovirulence-associated mycoviruses appears to be very important in use of the mycoviruses to control plant diseases [8,9]. Transmission of the mycoviruses occurs vertically to offspring in asexual or sexual spores, and horizontally between individuals by hyphae-hyphae fusion or hyphal anastomosis [11]. It seems that the DNA viruses differ from the RNA viruses in horizontal transmission. SsHADV-1 can be transmitted in two ways, namely hyphal anastomosis and contagious infection by the virus particles [10,12]. Moreover, Liu and colleagues [13] found that the mycophageous insect *Lycoriella ingenua* can act as a vector for transmission of SsHADV-1 from DT-8 to virulent strains of *S. sclerotiorum*. On the contrary, there is currently no good evidence for extracellular transmission of the RNA mycoviruses. Vegetative incompatibility between donor and recipient individuals usually blocks the horizontal transmission of the RNA viruses. It is actually a phenomenon of non-self allorecognition, which is characterized by formation of vacuoles, cytoplasm shrinkage, cell collapse and death [14]. Vegetative incompatibility is genetically controlled by heterokaryon genes (*het*) or vegetative incompatibility genes (*vic*) [15,16]. Two individuals sharing the same alleles at all *het* (or *vic*) loci are compatible and the RNA mycoviruses are transmissible between the two individuals [11]. In contrast, two individuals with different alleles at all or some *het* (or *vic*) loci are incompatible and the RNA mycoviruses can be blocked for the horizontal transmission. By disruption of the *vic* genes in *C. parasitica*, Zhang and Nuss [17] developed super CHV1-donor strains, which showed more efficient transmission of CHV1 than the wild-type CHV-donor strains. As a consequence, the genetically-modified strains were enhanced for suppression of chestnut blight, compared to the wild-type hypovirulent strains [17]. Recently, Wu and colleagues [18] reported that Sclerotinia sclerotiorum mycoreovirus 4 (SsMYRV4) can suppress the non-self-recognition in interaction between the SsMYRV4-donor strain and a mycelially incompatible mycovirus-free strain, thereby facilitating the horizontal transmission of other RNA viruses, such as Sclerotinia sclerotiorum debilitation-associated RNA virus (SsDRV).

Most mycoviruses have the genomes of RNA and a few have the genomes of DNA [1,7]. The mycoviruses in the families *Alphaflexiviridae*, *Gammaflexiviridea*, *Barnaviridae*, *Hypoviridae*, and *Narnaviridae* have the genomes of positive single-stranded RNA (+ssRNA). Recently, a few mycoviruses with the genomes of negative single-stranded RNA (−ssRNA) have been identified in *Botrytis cinerea* [19] and *Sclerotinia sclerotiorum* [20]. On the other hand, the mycoviruses in the families *Chrysoviridae*, *Endornaviridae*, *Megabirnaviridae*, *Patitiviridae*, *Quadriviridae*, *Reoviridae*, and *Totiviridae* have the genomes of double-stranded RNA (dsRNA). The members in families *Chrysoviridae*, *Megabirnaviridae*, *Patitiviridae*, *Quadriviridae*, *Reoviridae*, and *Totiviridae* produce coat proteins to accommodate their dsRNA genomes, whereas the members in *Endornaviridae* have no coat proteins and their dsRNA genomes are unencapsidated.

The endornaviruses were first reported in plants [21,22]. Later studies showed that they exist not only in plants [23], but also in fungi and oomycetes [24,25,26,27,28]. Each endornavirus has a non-segmented dsRNA genome, coding for a large polypeptide with the conserved RNA-dependent RNA polymerase domain [29]. Some endornaviruses have a site-specific nick in the positive RNA strand at the 5′-terminus [30,31].

Sclerotinia minor Jagger, a homothallic fungus, exhibits two mating type alleles (Inv+ and Inv−) that are mitotically stable [32] and are useful markers for tracking strains in special situations. *S. minor* is an important plant pathogenic fungus that infects many economically important crops, including Chinese cabbage (*Brassica rapa* subsp. *pekinensis*), lettuce (*Lactuca sativa*), oilseed rape (*Brassica napus*), peanut (*Arachis hypogea*), and sunflower [33,34,35,36]. It causes substantial economic losses in these crops in many countries, including Canada, China, and USA [37,38,39]. So far, management of *S. minor* on lettuce and peanut largely depends on application of fungicides, as highly resistant commercial cultivars against *S. minor* are not available in the two crops [40,41]. However, this chemical control often causes public concerns over the fungicide residues on plant products and environmental pollution. Therefore, it is imperative to develop alternative measures for control of *S. minor*, including biological control using mycoviruses.

Melzer and Boland [38] first reported transmissible dsRNA elements (possibly RNA viruses) in *S. minor*. However, none of the dsRNA elements in *S. minor* has ever been sequenced and identified. Our previous study identified a hypovirulent strain (LC22) of *S. minor*, which carries a dsRNA element of approximately 13 kb in size [39], but very little is known about the dsRNA element. This study was done to fulfill the following objectives: (i) to identify the dsRNA element in strain LC22 by cDNA cloning and sequence analysis; (ii) to characterize transmission of the dsRNA element among different strains of *S. minor*; and (iii) to evaluate the biocontrol potential of the dsRNA element.

## 2. Materials and Methods

### 2.1. Fungal Strains and Cultural Media

A total of 26 fungal strains were used in this study (Supporting Information Table S1). Twenty-six strains, representing 11 mycelial compatibility groups (MCGs), belong to *S. minor.* Twenty-three strains of *S. minor*, including LC22 and LC41, were isolated in 2012 from lettuce (*Lactuca sativa*) and a few weeds in Lichuan County and Xianning County of Hubei Province, China [38]. The remaining three isolates of *S. minor* (W1, W26, P13) were kindly provided by Dr. Barbara Shew of North Carolina State University in USA. Two cultural media, namely potato dextrose agar (PDA), and potato dextrose broth (PDB) were used in this study. Both PDA and PDB were prepared with peeled potato tubers.

### 2.2. Determination of Mycelial Growth Rate, Sclerotial Production, and Pathogenicity

Mycelial agar plugs of the strains were transferred to PDA in Petri dishes (9 cm diameter), one agar plug per dish and five dishes (replicates) for each strain. The diameter of each colony was measured at day 1 and day 2, and the diameter difference between the two measurements was used to calculate the radial mycelial growth rates [42]. These two measurements were used for growth rate calculation because some fast growing isolates could reach to the edge of the plates in three days. The cultures were further incubated for 20 days. Colony morphology was observed and number of the sclerotia formed in each dish was counted. The experiment was repeated one more time.

Pathogenicity of the strains was tested on detached leaves of oilseed rape (*Brassica napus* cultivar “Zhongshuang No. 9”) from 45-day-old plants. Mycelial agar plugs were inoculated on detached leaves placed on moisturized paper towels in plastic trays (45 × 30 × 2.5 cm, length × width × height), two agar plugs per leaf and 3 leaves (replicates) for each strain. The trays were individually covered with 1-mm-thick transparent films to maintain high humidity. Diameter of each necrotic lesion was measured two days after inoculation. The test was repeated once.

### 2.3. Extraction and Identification of dsRNA

DsRNA molecules in the mycelia were extracted, purified using the same procedures as those used in our previous studies [42,43,44,45]. Presence of dsRNA viruses was detected by agarose gel (1%, *w*/*v*) electrophoresis. The nature of the dsRNA was confirmed by digestion with RNase A (TaKaRa Biotechnology Co., Ltd., Dalian, China), RQ1 RNase-free DNase (Promega, Madison, WI, USA) and S1 nuclease (TaKaRa) [42,43]. The molecules that can be digested by RNase A, but not by DNase and S1 nuclease were considered to be dsRNA.

### 2.4. cDNA Cloning and Sequencing of SmEV1

The dsRNA was used as a template to generate cDNA fragments following the procedure used by Wu and colleagues [42,43,44]. The amplified DNA fragments were cloned, sequenced, and used to assemble the full-length cDNA sequence. Every base pair in the assembled sequence was ascertained by sequencing at least three clones.

### 2.5. Sequence Analysis

Open reading frame (ORF) in the full-length cDNA sequence of SmEV1 and polypeptides encoded by the ORF was deduced using the ORF Finder program in NCBI (http://www.ncbi.nlm.nih.gov/gorf/) with the standard codon usages. The sequences of previously reported endornaviruses and related outgroup viruses were retrieved from the NCBI GenBank database (http://www.ncbi.nlm.nih.gov/genomes) and used for comparative analysis. Multiple sequence alignment was carried out using DNAMAN software (V6.0, Lynnon Corporation, San Ramon, CA, USA) to determine the conserved motifs for the domains of MTR, Hel 1, and RdRp. Phylogenetic trees were constructed based on the amino acid sequences of MTR, Hel 1, and RdRp using the neighbor-joining (NJ) method in MEGA 5.0 [46] and tested with a bootstrap of 1000 replicates.

### 2.6. Northern Hybridization

For Northern blotting, 1 μg viral dsRNA was loaded in 1.2% (*w*/*v*) agarose gel in MOPS buffer (20 mM MOPS, 5 mM sodium acetate, 2 mM EDTA, pH 7.0) containing 2% formaldehyde (*v*/*v*). After electrophoresis, the gel was transferred to a nylon Zeta-Probe membrane (Bio-Rad, Hercules, CA, USA) by capillary blotting for 16 h using 20× SSC (3.0 M NaCl, 0.3 M sodium citrate, pH 7.0) as transfer buffer. Two probes, namely Probe 1 (800 bp long, nt 11335–12134) and Probe 2 (351 bp long, nt 40–390), were used in Northern hybridization. Two *E. coli* clones harboring the cDNA fragments of SmEV1 were used for generation of the probes by PCR with the primer pairs Probe 1F/1R and Probe 2F/2R (Supporting Information Figure S1, Table S2). The probes were labeled with digoxigenin (DIG). Northern blotting was done following the method described by Streit and colleagues [47]. The chemiluminescent signals in the probe DNA-RNA hybrids were detected using the reagents in the CDO-Star kit (GE Healthcare Life Sciences, Pittsburgh, PA, USA).

### 2.7. Horizontal Transmission of SmEV1 in S. minor

Strain LC22 of *S. minor* (MCG 5, *MAT* Inv+) was used as a donor of SmEV1 and 25 other strains of *S. minor* (MCGs 1 to 11) were used as recipients of SmEV1 (Table 1). Transmission of SmEV1 from strain LC22 to each of the recipient strains was tested using the pair culture technique described by Wu and colleagues [42,43,44]. The pair cultures on PDA were incubated at 20 °C for seven days. A mycelial agar plug from the margin area of the recipient colony distant from the inoculation point was transferred to a new PDA dish to establish a derivative of that recipient, designated by suffixing the recipient strain with a “V”. The transmission for each recipient strain was repeated three times. To ascertain that the derivative isolates were indeed due to transmission of SmEV1 from the donor strain LC22 to the recipient and not due to any possibility of contamination of LC22, the *MAT* alleles of the derivative isolates XN01V1, XN01V2, XN01V3, along with the donor strain LC22 (Inv+), and the recipient strain XN01 (Inv−), were detected by using PCR with the previously developed primers [32,39]. All of the 75 derivative isolates were individually tested for the presence of SmEV1 by RT-PCR using specific primer pair RdRp F/R (Supporting Information Table S2). Meanwhile, mycelial growth rate, sclerotial production, and pathogenicity of these derivatives were compared with their progenitors, using methods as described above.

### 2.8. Transmission of SmEV1 through Sclerotia

Sclerotia (a total of 120) of strain LC22 produced on PDA (20 °C, 30 days in the dark) were surface-sterilized with 0.1% HgCl_2_ solution (*w*/*v*) for 5 min, followed by rinsing for three times (1 min each) in sterilized water and individually transferred on PDA, one sclerotium per dish. The cultures were incubated at 20 °C for 10 days. The resulting single-sclerotium (SS) cultures were tested for pathogenicity on detached oilseed rape leaves (20 °C, 72 h). Eight SS isolates (S002, S004, S017, S038, S054, S085, S097, S104) were randomly selected and tested for present of SmEV1 by RT-PCR using the specific primer pair RdRp F/R (Supporting information Table S2), as well as for mycelial growth rates and sclerotial production on PDA at 20 °C. In these tests, LC22 and LC41 were used as controls.

### 2.9. Extraction of the Total RNA and RT-PCR Detection of SmEV1

Total RNA was extracted from 3-day-old mycelia (20 °C) of each strain or isolate using the TRIzol reagent (TaKaRa) following the manufacturer’s instructions. It was treated with DNase I to remove DNA contamination. Then, the extract was used as template for reverse transcription to synthesize cDNA using PrimeScript^®^ Reverse Transcriptase (TaKaRa) with the oligo (dT)_18_ primer. Finally, the cDNA was used as template in PCR for amplification of the RdRp region of SmEV1 with the primer pair RdRp F/R. Detection of the actin gene (*Actin*) by RT-PCR using the primer pair Actin qF2/qR4 (Supporting information Table S2) was used as control.

### 2.10. Biocontrol Assay

Mycelium of strain LC22 from five-day-old shake-culture (150 rpm, 20 °C) in PDB was collected by centrifugation at 5000 rpm for 10 min. The mycelial pellet was weighed and re-suspended in fresh PDB (3 g wet mycelial pellet in 100 mL PDB), and blended to generate a hyphal fragment suspension (HFS). Meanwhile, the detached leaves of oilseed rape were placed in two rows on moisturized paper towels in a plastic tray. The leaves in one row were treated with the HFS at 500 μL HFS per leaf. Then, the leaves in the other row were treated with fresh PDB (control) also at 500 μL per leaf. The leaves were inoculated with the mycelial agar plugs from a two-day-old PDA culture of strain LC41 (20 °C), one agar plug per leaf. The tray was covered with a 1-mm-transparent plastic film to maintain a high level of humidity. It was then placed in a growth chamber (20 °C, 12 h light/12 h dark). The lesion diameter around each inoculated mycelial agar plug was measured at 48, 72, and 96 h after inoculation. The experiment was repeated three times.

### 2.11. Data Analysis

The procedure UNIVARIATE in the SAS software (SAS Institute, Cary, NC, USA, v. 8.0, 1999) was used to analyze the data on mycelial growth rates, number of sclerotia per dish, sclerotium weight, and leaf lesion diameters for strain LC22, the SmEV1-transfected recipient derivatives and their parental strains in related comparative assays. Data on each parameter between strains LC22 and LC41, between each SmEV1-transfected recipient derivative and its parental recipient were compared using Student’s *t* test at *p* < 0.05 or *p* < 0.01. Meanwhile, the procedure ANOVA (analysis of variance) in the SAS software was used to analyze the data on mycelial growth rates and number of sclerotia per dish produced by LC22, eight representative single-sclerotium isolates of LC22 and LC41. Means of each parameter among the isolate/strains were separated using least significant difference (LSD) test at *p* < 0.05.

## 3. Results

### 3.1. Cultural Characteristics and Pathogenicity of Strain LC22

**Figure 1:**
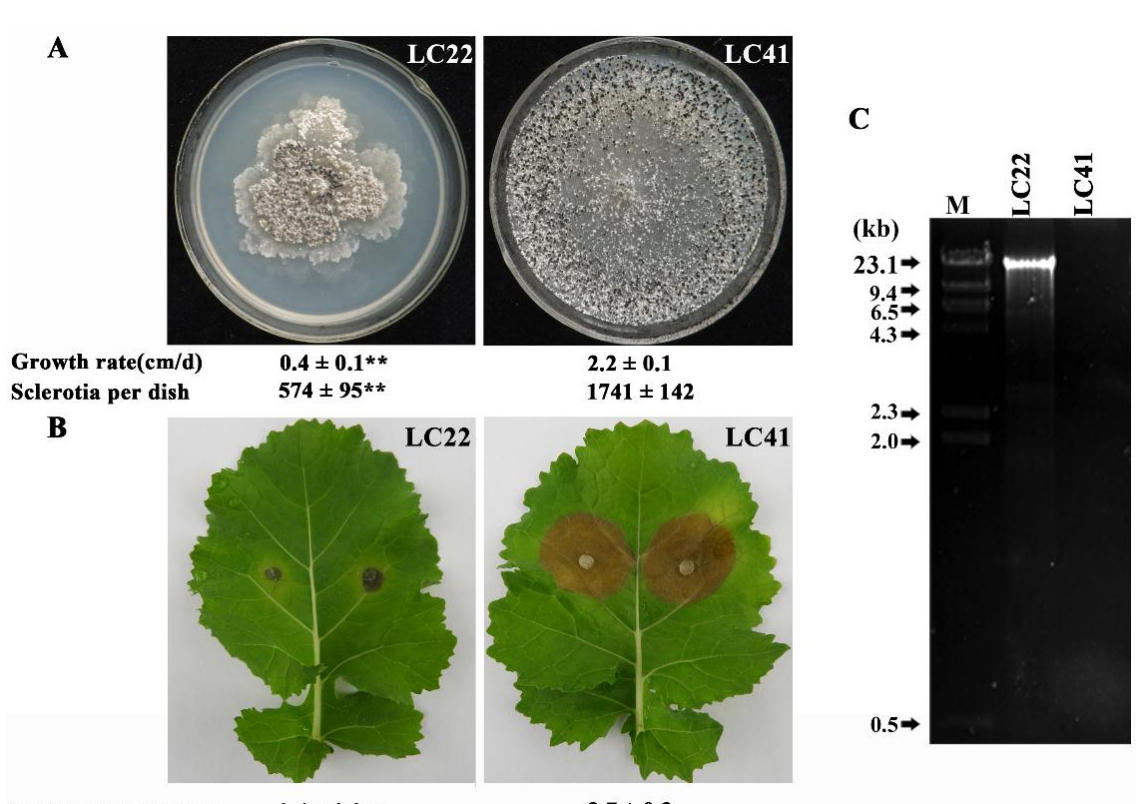
Comparison of strains LC22 and LC41 of Sclerotinia minor in mycelial growth rate, sclerotial production, pathogenicity and presence/absence of dsRNA. (**A**) Colony morphology, growth rate and sclerotial formation on potato dextrose agar (20 °C, 10 d). ** *p* < 0.01 between LC22 and LC41, Student’s *t* test; (**B**) Lesions on oilseed rape leaves two days after inoculation (20 °C). ** *p* < 0.01 between LC22 and LC41, Student’s *t* test; (**C**) An agarose gel electrophoregram showing the band of the dsRNA extracted from the mycelia of LC22 and LC41. The extracts were treated with S1 nuclease and DNase I before being loaded in the gel for electrophoresis. The DNA marker is *λ*DNA/*Hind* III.

Strain LC22 of *S. minor* grew on PDA significantly slower (*p* < 0.01) than that of strain LC41 of *S. minor* (Figure 1A). Strain LC22 formed colonies with abnormal morphology characterized by producing numerous irregular mycelial sectors in the colony margin. In contrast, LC41 colonized the entire PDA dishes after 3 days without producing any irregular mycelial sectors. LC22 formed fewer sclerotia in 10-day-old PDA cultures (574 sclerotia/dish) than LC41 (1741 sclerotia/dish). Furthermore, strain LC22 was less virulent on detached oilseed rape leaves (Figure 1B). Therefore, compared to LC41, LC22 is attenuated in mycelial growth, sclerotial production, and pathogenicity (or aggressiveness). It is a hypovirulent strain in terms of pathogenicity.

### 3.2. DsRNA in Strain LC22 and Its Mycoviral Nature

**Figure 2:**
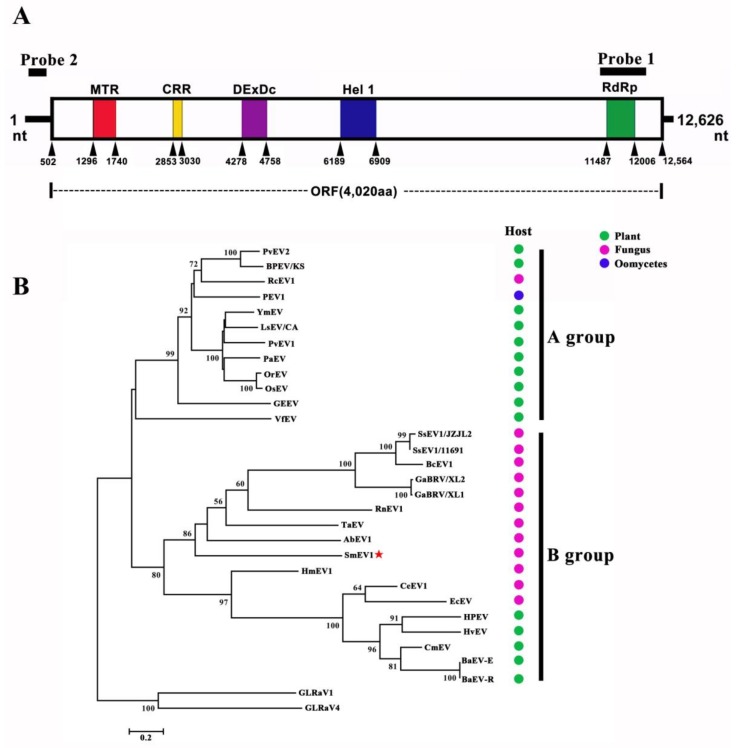
Genome characteristics and phylogeny of SmEV1. (**A**) A schematic diagram showing the genome organization of SmEV1. The coding stand of SmEV1 comprises one large open reading frame (ORF) coding for a polyprotein with the conserved domains of methyltransferase (MTR), cysteine-rich region (CRR), DEXDc, helicase 1 (Hel 1) and RNA-dependent RNA polymerase superfamily_2 (RdRp); (**B**) A maximum-likelihood tree showing the phylogenetic relationship of SmEV1 with 28 other endornaviruses. Grapevine leafroll-associated virus 1 and 4 (GLRaV1 and GLRaV4, GenBank Acc. Nos. ANP22157.1 and NC_016416.1, respectively) were used as outgroups. The tree was inferred from the RNA-dependent RNA polymerase (RdRp) domain. See Table 2 for abbreviation of the endornaviruses. Bootstrap support values lower than 50% are not shown.

A dsRNA element of ~13 kb in size was detected in the mycelia of LC22, but was not detected in the mycelia of LC41 or any other of the 24 *S. minor* strains (Figure 1C; Supporting information Table S1). The full-length cDNA sequence of 12,626 bps for the dsRNA in LC22 was finally obtained and deposited in the GenBank under the accession number MG255170. The cDNA sequence has the GC content of 47.4%. It was deduced to have one large open reading frame (ORF) starting from nt 502 to nt 12,564 and two untranslated regions (UTR) at 3′- and 5′-termini with the length of 62 and 502 bp, respectively (Figure 2A). The ORF codes for a polyprotein containing 4020 amino acids (aa) with a calculated molecular mass of 448 kDa. BLAST search indicated that the polyprotein contains a viral methyltransferase (MTR) domain, a cysteine-rich region (CRR), two putative viral helicase domains (DExDc and Hel 1) and a domain for RNA-dependent RNA polymerase_2 (RdRp_2) (Figure 2A; Supporting information Figures S3–S6). This genome structure is similar to that of the previously-reported endornaviruses [1]. Therefore, the dsRNA in LC22 represents the genome of an endornavirus, designated thereafter as Sclerotinia minor endornavirus 1/LC22 (SmEV1/LC22). Northern blotting result showed that both probes detected only one dsRNA band (Supporting Information Figure S2), suggesting that no nick exists in SmEV1.

### 3.3. Phylogeny of SmEV1/LC22

SmEV1/LC22 was compared with 29 other endornaviruses in homology of the whole polyprotein and each of the three conserved domains (MTR, Hel 1, RdRp_2). SmEV1/LC22 is identical by 7.60–21.99%, 13.87–34.62%, 15.48–30.20%, and 23.92–47.43% to the other endornaviruses in the whole polyprotein, MTR, Hel 1 and RdRp_2, respectively (Table 2). Phylogenetic analyses were done based on the conserved domains of RdRp_2, MTR, and Hel 1. The endornaviruses formed two groups (A and B). SmEV1 is located in B group in all the three phylogenetic trees. It is most closely related to Sclerotinia sclerotiorum endornavirus 1 (SsEV1), Botrytis cinerea endornavirus 1 (BcEV1), Gremmeniella abietina type B RNA virus (GaBRV), Rosellinia necatrix endornavirus 1 (RnEV1), Tuber aestivum endornavirus (TaEV), and Alternaria brassicicola endornavirus 1 (AbEV1) (Figure 2; Supporting Information Figures S3–S6). Based on currently valid species demarcation criteria for the family *Endornaviridae* (i.e., isolated from a different host species, and less than 75% sequence identity) [48], SmEV1/LC22 should be considered a novel species in the genus *Endornavirus*.

### 3.4. Horizontal Transmission of SmEV1

**Figure 3:**
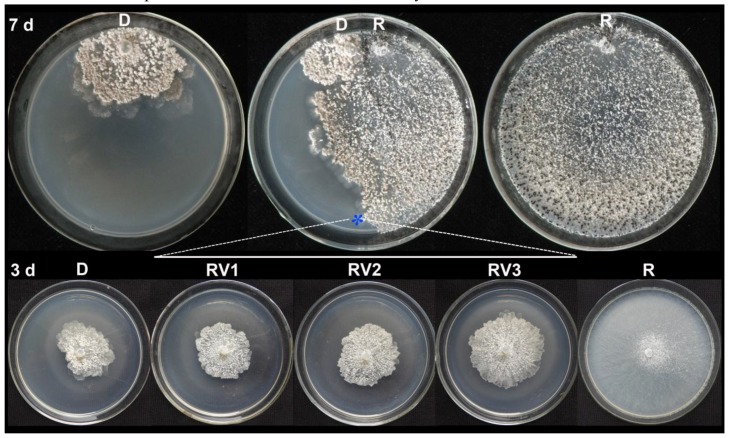
Horizontal transmission of SmEV1 from the donor strain LC22 (D, donor; SmEV1^+^) to the recipient strain LC41 (R, recipient; SmEV1^−^) through hyphal contact in a pair culture, with comparison of the strains in single cultures. Top row: two single cultures of the donor strain LC22 and the recipient strain LC41, respectively, and a pair culture of LC22/LC41 (D/R) on PDA (20 °C, seven days). “*” in the pair-culture indicates the area where a mycelial agar plug was removed for generating a derivative of the recipient strain designated as RV. Bottom row, PDA cultures (20 °C, three days) of the donor strain LC22 (D, SmEV1^+^) and the recipient strain LC41 (R, SmEV1^−^), and three derivative isolates generated from the recipient strain LC41 in the pair cultures of LC22/LC41 (D/R).

Results of the transmission experiments showed that all the recipient-derived strains exhibited attenuated mycelial growth and formation of numerous irregular mycelial sectors in the colony margin at 7 days after pair culturing at 20 °C with the donor strain LC22 on PDA (Figure 3). In order to ascertain the origin of the derivatives from the recipients, not from the donor (LC22), three derivatives, namely XN01V1, XN01V2 and XN01V3, were selected and compared with XN01 and LC22 in the *MAT* alleles (Inv+ and Inv−), which were detected by PCR using specific primers MAT1-1-F/MAT1-1-R (for Inv−) and Type IIF/Type-IIR (for Inv+) [32]. Results showed that XN01V1, XN01V2 and XN01V3 had the same *MAT* allele as XN01 (Inv−), whereas LC22 had the *MAT* allele of Inv+ (Supporting information Figure S7).

All the 25 derivatives were positively for SmEV1 accumulation (Table 1). They were significantly (*p* < 0.01) suppressed for mycelial growth and sclerotial production on PDA, and for infection of leaves of oilseed rape, compared to the virus-free recipient strain (Table 1). Therefore, hypovirulence and decline characteristics of strain LC22 and SmEV1 were successfully transmitted by hyphal contact to isolates of different vegetative compatibility groups of *S. minor.* These results indicated that SmEV1 has a wide spectrum of horizontal transmissibility.

### 3.5. Transmission of SmEV1 through Sclerotia

**Figure 4:**
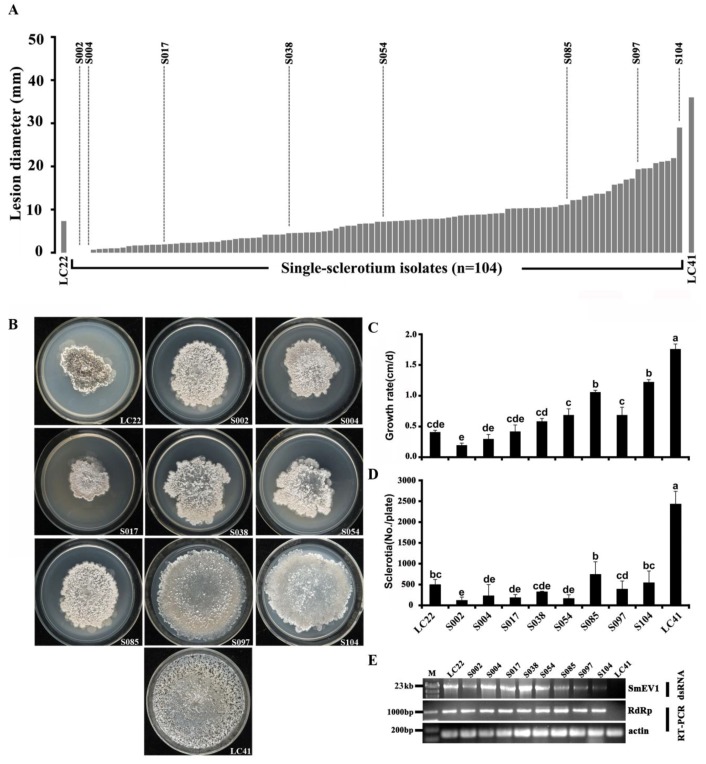
Transmission of SmEV1 in strain LC22 of Sclerotinia minor through sclerotia. (**A**) A histograph showing the average lesion diameters caused by the hypovirulent strain LC22, 104 single-sclerotium (SS) isolates of strain LC22 and the virulent strain LC41 on detached leaves of oilseed rape (20 °C, 72 h). Note reduced average leaf lesion diameters caused by strain LC22 and its SS progenies, compared to the lesion caused by LC41. Eight representative SS isolates selected for further analyses were labeled in the graph; (**B**) Colony morphology of LC22, eight SS isolates and LC41 (PDA, 20 °C, 30 d); (**C**) Average growth rates (*n* = 5) for LC22, eight SS isolates and LC41. Different letters on the bars in each graph indicate significant difference (*p* < 0.05) according to the Least Significant Difference test; (**D**) Average sclerotial yield (*n* = 5) produced by LC22, eight SS isolates and strain LC41 in 30-day-old PDA cultures; (**E**) Detection of SmEV1 in LC22, eight SS isolates and LC41 by dsRNA extraction and RT-PCR.

Sclerotia produced by *Sclerotinia* species play an important role (survival and reproduction) in their life cycle. Sclerotia of *S. minor* can survive the harsh summer and winter seasons and they usually germinate to produce mycelia, whereas occasionally germinate to produce ascospores [36]. Therefore, transmission of SmEV1 from mycelia to sclerotia is important in terms of biological control using SmEV1. A total of 104 single-sclerotium (SS) isolates were obtained from the 120 sclerotia of strain LC22 formed in PDA cultures. Results showed that the leaf lesion diameters caused by these SS isolates ranged from 0.0 to 2.9 cm, significantly smaller than that of 3.6 cm caused by LC41 (Figure 4A). Eight SS isolates (S002, S004, S017, S038, S054, S085, S097, S104) were randomly selected for determining mycelial growth rates and sclerotial formation on PDA (20 °C) and for the presence of SmEV1 by dsRNA profiling and RT-PCR. All these SS isolates formed colonies with the abnormal morphology (irregular sectors in the colony margin), compared to the colonies formed by LC41 (Figure 4B). They grew at slower growth rates (0.2 to 1.2 cm/d) than LC41 (1.8 cm/d) (Figure 4C) and formed fewer sclerotia (118 to 743 sclerotia/dish) than LC41 (2430 sclerotia/dish) (Figure 4D). SmEV1 was detected in mycelia of each of these SS isolates (Figure 4E). These results suggested that SmEV1 could be transmitted vertically through sclerotia.

### 3.6. Biocontrol Efficacy of SmEV1

**Figure 5:**
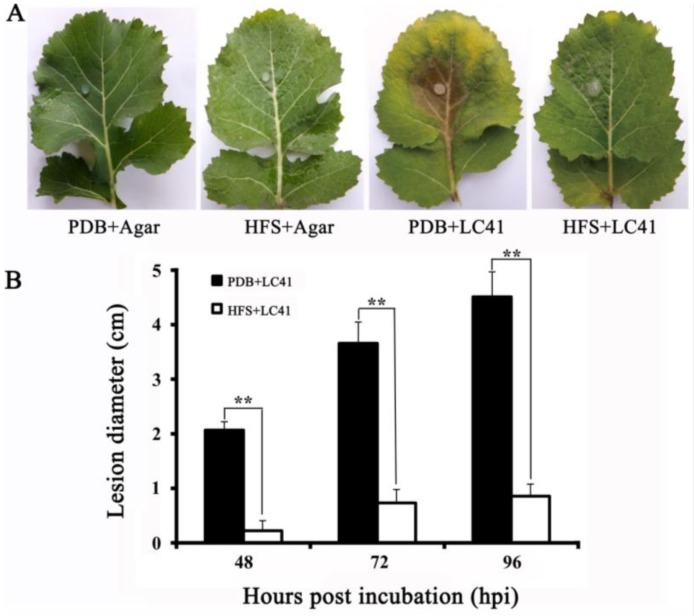
Effect of pre-treatment of oilseed rape leaves with the hyphal fragment suspension (HFS) of the hypovirulent strain LC22 of Sclerotinia minor on infection by the virulent strain LC41 of *S. minor*. (**A**) Lesions caused by strain LC41 at 96 h post inoculation (20 °C) on leaves of different treatments. PDB + Agar, the leaf was pre-treated with potato dextrose broth (PDB) alone and challenge-inoculated with a fresh PDA plug; HFS + Agar, the leaf was pre-treated with the HFS of LC22 alone and challenge-inoculated with a fresh PDA agar; PDB + LC41, the leaf was pre-treated with PDB alone and challenge-inoculated with a mycelial agar plug of LC41; HFS + LC41, the leaf was pre-treated with the HFS of LC22 and challenge-inoculated with a mycelial agar plug of LC41; (**B**) A histograph showing average leaf lesion diameters (*n* = 18) of different treatments. ** *p* < 0.01, Student’s *t* test.

An indoor assay on leaves of oilseed rape was done to test efficacy of pre-treatment with the mycelial fragments of LC22 in suppression of the infection by challenge-inoculated virulent strain LC41 (Figure 5A). On the leaves of the control treatment (PDB alone), strain LC41 caused severe infection on the leaves (Figure 5B). In contrast, on the leaves pre-treated with the hyphal fragments of strain LC22 alone without inoculation with strain LC41, no visible symptoms were observed. On the leaves pre-treated with the hyphal fragments of strain LC22 and challenge-inoculated with LC41, only slight infection was observed with formation of tiny restricted necrotic lesions or spots (Figure 5B). Additionally, leaf tissues with the lesions in the LC22 HFS-treated leaves were cut off, surface-sterilized with NaOCl and incubated on PDA for isolation of the fungus in the lesions. Five fungal isolates were obtained. These isolates grew on PDA (20 °C) and formed the colonies with the abnormal colony morphology similar to the colonies formed by LC22. Therefore, LC22 can effectively suppress infection of leaves of oilseed rape by virulent *S. minor*.

## 4. Discussion

In this study, we characterized a novel mycovirus (SmEV1) in a hypovirulent strain LC22 of *S. minor*. Based on phylogenetic analysis of RdRp and genome organization, SmEV1 belongs to the subclade B group in the clade of endornaviruses. The SmEV1 horizontal transmission experiments showed that hypovirulence and associated decline characteristics of strain LC22 and SmEV1 were successfully cotransmitted to strains belonging to different vegetative compatibility groups of *S. minor.* As far as we know, this is the first report of a pathogenicity debilitation-associated endornavirus in *S. minor*. With one exception of Helicobasidium mompa endornavirus 1 (HmEV-1) [28], endornavirus infection usually does not cause any visible abnormal symptoms for host fungi [26]. SmEV1 associated debilitation symptoms are similar to previous studies of hypovirulence caused by other mycoviruses in strains of *S. sclerotiorum* [12,49,50,51] and other plant pathogenic fungi [8,52,53,54]. Although SmEV1 cannot be tested for virion transfection without virus particles, the infectious viral cDNA method [55] might be used to determine the causal relationship between SmEV1 infection and host hypovirulence in future studies.

So far, 29 *Endornavirus* genomes have been fully sequenced and characterized, 15 infecting plants, 13 infecting fungi, and one infecting oomycetes (Table 2). One feature of most endornaviruses is that there is a site-specific nick in the coding strand at 5′ terminus [48]. In previous reports, 15 endornaviruses were investigated for presence or absence of the nick in the coding strand. Nine endornaviruses belonging to *Alphaendornavirus* have the nick in the coding strand [24,28,31,53,54,55,56,57,58,59]. Other six endornaviruses infecting fungi do not have the site-specific nicks in its dsRNA genomes [25,26,27,60,61], five of them belong to *Betaendornavirus*, with the exception of Rhizoctonia cerealis endornavirus 1 (RcEV1), which belongs to *Alphaendornavirus*. Our results also showed that SmEV1 in *Betaendornavirus* does not have a nick in 5′-terminus of the coding strand. However, the biological significance of the site-specific nicks in any of these endornaviruses remains unknown. They may play roles in regulation of replication or transcription of endornaviruses.

Endornaviruses usually cause a persistent infection in hosts [23,29]. The endornavirus members in plant can transmit vertically by pollen grains and seeds, but can hardly transmit by contact [62]. In fungi, only one endornavirus (i.e., HmEV1-670) was reported to be able to transmit through hyphal anastomosis [63]. In our study, hyphal incompatibility did not restrict horizontal transmission of SmEV1 in *S. minor*. Previous studies have shown that the two mycoviruses (SsHADV-1 and SsPV1) could easily and efficiently transmit between two vegetative incompatible strains in *S. sclerotiorum* [12,51]. Furthermore, SsMYRV4 was found capable of suppression of vegetative incompatibility-mediated programmed cell death (PCD), thus facilitating horizontal transmission of other mycoviruses among vegetative incompatible strains of *S. sclerotiorum* [18]. Whether the horizontal transmission of SmEV1 is achieved by inhibiting vegetative incompatibility reaction remains unknown and needs to be further characterized. Another possible explanation is that the vegetative incompatibility reaction in *S. minor* is not strong enough to restrict the horizontal transmission of SmEV1.

SmEV1 persistently exist in its hosts. This phenomenon is similar to that reported for other endornaviruses [64] and some other mycoviruses, such as SsPV1 [51], and BcRV1 [45]. We had tried to eliminate SmEV1 from strain LC22 using different methods, including hyphal-tip culturing, protoplast regeneration and plant-inoculation and re-isolation. However, all the methods failed to generate a virus-free strain. Further studies are warranted on curing *S. minor* strain LC22 of SmEV1 by single ascospore isolation. Our previous study has demonstrated that the population characteristics of *S. minor* were simple with a few mycelial compatibility groups [39]. *S. minor* usually germinate myceliogenically. Persistence of SmEV1 in mycelia and sclerotia might be useful for future exploitation of SmEV1 to control sclerotinia diseases caused by *S. minor*. The ability of the hypovirulent strain LC22 in suppressing plant infection by virulent strain LC41 of *S. minor* could be due to SmEV1 horizontal transmission via hyphal anastomosis from LC22 to LC41 and caused hypovirulence. Another reason may be the hypovirulent strain LC22 induces plant resistance to the virulent strain LC41 of *S. minor*.

In conclusion, we isolated and characterized a novel pathogenicity debilitation-associated endornavirus (SmEV1) in *S. minor*, and demonstrated SmEV1 has a wide spectrum of transmissibility. SmEV1 has the ability to spread horizontally among isolates regardless of vegetative incompatibility barriers between different VCGs in *S. minor.* The hypovirulent strain LC22 has a protective activity against attack by virulent *S. minor* according to the detached leaf assays in this study. Further evaluation of using hypha and sclerotia to transmit SmEV1 as a biological control agent to control *S. minor* under field conditions is needed.

## Figures and Tables

**Table 1:** Endornavirus SmEV1 transmission from the donor strain LC22 (MCG 5) to other strains of Sclerotinia minor (MCGs 1 to MCG 11), and effect of SmEV1 introduction on mycelial growth, sclerotial yield, and pathogenicity of the recipients.

Recipient Strain (MCG)	SmEV1 ^1^		Growth Rate (cm/d) ^2^		Sclerotia Per Dish ^2^		Leaf Lesion Diameter (cm) ^3^	
	Before	After	Before	After	Before	After	Before	After
LC53 (MCG1)	−	+	1.8	0.6 **	1806	1259 **	4.5	1.6 **
XN19 (MCG1)	−	+	1.8	0.7 **	2286	835 **	3.3	2.2 **
XN21 (MCG1)	−	+	1.9	0.5 **	1881	674 **	3.6	0.4 **
XN35 (MCG1)	−	+	1.6	0.2 **	2550	1200 *	2.9	0.5 **
DY02 (MCG2)	−	+	1.5	1.0 **	1978	1411 **	3.7	1.6 **
LC02 (MCG2)	−	+	1.7	1.1 **	2300	843 **	3.6	1.6 **
LC20 (MCG2)	−	+	1.5	0.7 **	1719	734 **	3.1	1.0 **
LC28 (MCG2)	−	+	1.4	1.1 **	2416	1278 *	3.8	0.9 **
LC41 (MCG2)	−	+	1.8	1.2 **	2198	1196 **	3.2	0.5 **
LC11 (MCG3)	−	+	1.6	0.3 **	2224	575 **	3.3	1.0 **
LC19 (MCG3)	−	+	1.7	0.2 **	1742	1014 **	3.2	0.6 **
LC46 (MCG3)	−	+	1.7	0.9 **	2432	889 **	2.6	0.8 **
XN01 (MCG3)	−	+	1.8	0.8 **	2880	1673 **	2.5	0.6 **
XN12 (MCG4)	−	+	2.0	1.2 **	2792	1408 **	3.9	0.2 **
XN13 (MCG4)	−	+	1.6	1.1 **	2556	1000 **	3.5	2.4 **
LC15 (MCG5)	−	+	1.8	1.4 **	2316	1475 **	3.4	1.3 **
LC38 (MCG5)	−	+	1.3	0.3 **	1188	674 **	0.8	0.2 **
XN14 (MCG6)	−	+	1.7	1.4 **	2350	1225 **	2.2	1.3 **
XN34 (MCG6)	−	+	1.8	0.4 **	1661	1124 *	4.0	0.7 **
LC29 (MCG7)	−	+	2.0	0.8 **	2492	1105 **	3.8	0.7 **
LC47 (MCG7)	−	+	2.1	1.2 **	2459	1472 **	3.5	1.1 **
LC36 (MCG8)	−	+	1.5	0.6 **	2691	1152 **	2.9	2.1 **
P13 (MCG9)	−	+	1.9	0.3 **	1029	528 **	3.5	0.6 **
W1 (MCG10)	−	+	1.7	0.2 **	1788	782 **	3.7	0.7 **
W26 (MCG11)	−	+	1.6	0.3 **	1230	956 **	3.4	0.7 **

^1^ Based on detection of SmEV1 RdRp domain using RT-PCR tests for each strain three times with independent total RNA extractions. “+” and “−” indicate presence and absence of SmEV1, respectively; “*” and “**” indicate significant difference at *p* < 0.05 and *p* < 0.01, respectively, according to Student’s *t* test. ^2^ Average growth rate and the sclerotial yield values for each *S. minor* strain of 10 replicates (two tests each with 5 replicates) on PDA at 20 °C in Petri dishes (9 cm in diameter). ^3^ Average of lesion diameter of detached leaves assays of oilseed rape with 12 replicates (two tests each with three leaves, each leaf with two inoculation sites).

**Table 2:** Percent identity of amino acid sequences between SmEV1 and other endornaviruses determined by multiple alignments of the full-length polyprotein sequence and the conserved domains coding for methyltransferase (MTR), helicase 1 (Hel 1), RNA-dependent RNA polymerase (RdRp).

Virus	Host	Genome Length	Identity (%)				Acc. No.	Presence of Nick
			ORF	MTR	Hel	RdRp		
Botrytis cinerea endornavirus 1 (BcEV1)	F	11,557 bp	20.37	33.33	24.39	45.45	KU923747	−
Alternaria brassicicola endornavirus 1 (AbEV1)	F	10,290 bp	15.09	30.90	24.28	32.41	NC_026136	ND
Tuber aestivum endornavirus (TaEV)	F	9760 bp	15.87	34.62	−	41.18	NC_014904	ND
Sclerotinia sclerotiorum endornavirus 1/JZJL2 (SsEV1/JZJL2)	F	10,770 bp	21.81	33.33	23.48	47.43	NC_021706	−
Sclerotinia sclerotiorum endornavirus 1/11691 (SsEV1/11691)	F	10,513 bp	21.53	32.90	23.89	47.04	NC_023893	−
Gremmeniella abietina type B RNA virus XL2 (GaBRV/XL2)	F	10,374 bp	21.72	33.19	30.20	46.64	DQ399290	−
Gremmeniella abietina type B RNA virus XL1 (GaBRV/XL1)	F	10,375 bp	21.99	33.19	29.80	45.45	NC_007920	−
Rhizoctonia cerealis endornavirus 1 (RcEV1)	F	17,486 bp	9.29	15.15	17.74	25.39	NC_022619	−
Rhizoctonia solani endornavirus RS002 (RsEV/RS002)	F	14,694 bp	9.78	18.10	18.43	−	KC792590	ND
Yerba mate endornavirus (YmEV)	P	13,954 bp	11.67	−	17.32	26.56	NC_024455	ND
Persea americana endornavirus (PaEV)	P	13,459 bp	10.75	−	15.48	24.22	NC_016648	ND
Oryza rufipogon endornavirus (OrEV)	P	13,936 bp	11.71	−	15.69	25.00	NC_007649	+
Bell pepper endornavirus (BPEV)	P	14,728 bp	12.01	17.32	17.74	27.73	NC_015781	ND
Oryza sativa endornavirus (OsEV)	P	13,952 bp	18.68	−	17.65	24.61	D32136	+
Vicia faba endornavirus (VfEV)	P	17,635 bp	8.91	−	15.63	26.95	AJ000929	+
Phytophthora endornavirus 1 (PEV1)	O	13,883 bp	12.59	−	16.47	26.95	AJ877914	+
Lagenaria siceraria endornavirus-California (LsEV-CA)	P	15,088 bp	10.04	−	−	23.92	NC_023641	ND
Phaseolus vulgaris endornavirus 1 (PvEV1)	P	13,908 bp	11.99	−	20.00	26.17	AB719397	+
Phaseolus vulgaris endornavirus 2 (PvEV2)	P	14,820 bp	11.16	19.05	20.40	26.56	AB719398	+
Helicobasidium mompa endornavirus 1 (HmEV1)	F	16,614 bp	9.88	−	17.94	26.07	AB218287	+
Grapevine endophyte endornavirus (GEEV)	P	12,154 bp	11.89	−	17.53	24.71	NC_019493	ND
Chalara endornavirus (CeEV1)	F	11,602 bp	12.48	−	19.22	26.95	GQ494150	ND
Basella alba endornavirus-Eclipse (BaEV-E)	P	14,027 bp	7.86	−	18.50	24.61	AB844264	+
Basella alba endornavirus-Rubra (BaEV-R)	P	14,027 bp	7.86	−	18.50	24.22	AB844265	+
Erysiphe cichoracearum endornavirus (EcEV)	F	11,908 bp	8.71	−	18.18	24.61	KT38810	ND
Rosellinia necatrix endornavirus (RnEV1)	F	9639 bp	14.45	24.03	27.98	37.65	LC076696	−
Hordeum vulgare endornavirus (HvEV)	P	14,243 bp	7.60	−	18.55	25.78	KT721705	ND
Cucumis melo endornavirus (CmEV)	P	15,078 bp	9.33	−	−	25.10	KT727022	ND
Hot pepper endornavirus (HPEV)	P	14,729 bp	8.6	13.87	−	26.95	JN019858	+

Note: F = Fungus, P = Plant, O = Oomycete; ORF = Open reading frame; MTR = Methyltransferase, Hel = Viral RNA helicase, RdRp = RNA-dependent RNA polymerase; “–” = domain not present or no nick; “+” = nicked, ND = not determined.

## Supplementary Materials

The following are available online at http://www.mdpi.com/1999-4915/10/11/589/s1, Figure S1. A schematic diagram showing the strategy used for full-length cDNA cloning of the SmEV1 genome. The PCR primers and the 3’-adaptor used for the cDNA cloning are shown. See Table S2 for the oligonucleotide sequences of the primers/adaptor; Figure S2. (A) A schematic diagram showing positions of Probe 1 and Probe 2 used in Northern blotting; (B) Banding pattern of SmEV1 dsRNA (left), and Northern blotting with the two DNA probes (right); Figure S3. (A) Multiple alignment of the amino acid sequences of the methyltransferase (MTR) domain of SmEV1 and other endornaviruses. “I” to “IV” represent motifs I to IV. “*” and “.” represent identical and chemically-similar amino acids, respectively; (B) A maximum-likelihood tree showing the phylogeny of SmEV1 and other endornaviruses. The tree was inferred from the MTR domain. Rehmannia mosaic virus (RMV, GenBank acc. no. JX575184), ngewotan virus (NV, GenBank acc. no. AFY98072.1) and plum bark necrosis stem pitting-associated virus (PBNSPaV, GenBank acc. no. CDM63857.1) were used as outgroups. See Table 1 for abbreviations of the endornaviruses in the phylogenetic tree. Bootstrap support values lower than 50% are not shown; Figure S4. Multiple alignment of the amino acid sequences of the cysteine-rich region (CRR) of SmEV1 and other endornaviruses. Cysteine in the sequences were highlighted; Figure S5. (A) Multiple alignment of the amino acid sequences of the helicase 1 domain (Hel 1) of SmEV1 and other endornaviruses. “I” to “VI” represent motifs I to VI. “*” and “.” represent the identical and chemically similar amino acids, respectively. (B) Phylogeneny of SmEV1 and other endornaviruses based on the Hel 1 domain. Grapevine leafroll-associated virus 1 (GLRaV1, GenBank acc. no. ANP22157.1) was used as outgroup. See Table 1 for abbreviation of the virus. Bootstrap support values lower than 50% are not shown; Figure S6. Multiple alignment of the amino acid sequences of the domain for RNA-dependent RNA polymerase superfamily_2 (RdRp) of SmEV1 and other endornaviruses. “I” to “VIII” represent motifs I to VIII. “*” and “.” represent the identical and chemically similar amino acids, respectively; Figure S7. Agarose gel electrophoregrams showing the genetic markers for the *MAT* alleles (Inv− and Inv+) in the donor strain LC22, the recipient strain XN01, and the three derivative isolates XN01V1, XN01V2, and XN01V3. M, DNA marker; ITS, Internal Transcribed Spacer, Table S1. List of Sclerotinia minor strains used in the study; Table S2. Primers/adaptor used in this study and their oligonucleotide sequences.
